# New insights into M1/M2 macrophages: key modulators in cancer progression

**DOI:** 10.1186/s12935-021-02089-2

**Published:** 2021-07-21

**Authors:** Jiuyang Liu, Xiafei Geng, Jinxuan Hou, Gaosong Wu

**Affiliations:** 1 Department of Thyroid and Breast Surgery, Zhongnan Hospital of Wuhan University, Wuhan, China; 2 Department of Ultrasound Imaging, Hubei Cancer Hospital, Wuhan, China

**Keywords:** Tumor-associated macrophages, Cancer, Metastasis, Polarization

## Abstract

Infiltration of macrophages in and around tumor nest represents one of the most crucial hallmarks during tumor progression. The mutual interactions with tumor cells and stromal microenvironment contribute to phenotypically polarization of tumor associated macrophages. Macrophages consist of at least two subgroups, M1 and M2. M1 phenotype macrophages are tumor-resistant due to intrinsic phagocytosis and enhanced antitumor inflammatory reactions. Contrastingly, M2 are endowed with a repertoire of tumor-promoting capabilities involving immuno-suppression, angiogenesis and neovascularization, as well as stromal activation and remodeling. The functional signature of M2 incorporates location-related, mutually connected, and cascade-like reactions, thereby accelerating paces of tumor aggressiveness and metastasis. In this review, mechanisms underlying the distinct functional characterization of M1 and M2 macrophages are demonstrated to make sense of M1 and M2 as key regulators during cancer progression.

## Introduction

Tumor, to some extent, could be defined as a systemic disease of immuno-imbalance [1]. Malignant tumor is characterized by uncontrolled cell proliferation due to the unbalance between the mutations of oncogenes and tumor suppressor genes [2]. Once the mutation has been detected by host immune system, various lymphocytic infiltrates would accumulate in and around tumor zones to harbor intrinsic and adaptive immunities [3]. In this case, the tumoral immune microenvironment plays crucial roles during the multi-stage processes of tumorigenesis and progression.

Within the immune microenvironment, resident and recruited macrophages act as first lines of immunoregulatory functions and key modulators during tumor progression. In response to tumor-derived or microenvironmental signals, macrophages undergo phenotypically polarization to a universe of activation states [4]. Extremely, the macrophages plasticity could be summarized as M1 (classically activated) and M2 (alternatively activated) phenotypes, for better understandings of their distinct cellular and molecular mechanisms [5]. Both of M1 and M2 macrophages are involved in the affection of tumor-related inflammatory [6], whereas M2 is prone to promote angiogenesis and neovascularization, as well as stromal activation and remolding [7, 8], thereby impacting cancer progression positively and patient’ prognosis negatively [9]. Consequently, the interplay between host immune system and tumor cells could be representatively indicated from the aspect of M1/M2 macrophages.

For long momentum studies have emphasized the significance of M1/M2 macrophages. Some questions remain to be debated. Whether phenotypical polarizations explain pleiotropic but opposed activities of TAM? To what extent M1 and M2 differ considering functional properties under the framework of tumor microenvironment? Do spatial locations of macrophages especially M2 contribute largely to tumor progression? After describing the origination and differentiation of macrophages, this review would focus on the distinct functional characteristics of M1/M2 in terms of three major aspects, including immunoediting, angiogenesis and neovascularization, and stromal orchestration.

### Origination and differentiation of macrophages

#### Origination and maturation of macrophages

Macrophages populated within tissues are grouped resident macrophages and recruited macrophages. For long resident macrophages are proved to be replaced by migrated circulating peripheral blood monocytes, which acted as precursors of macrophages [5]. Recent studies have found a fact that resident macrophages are endowed with self-renewal capacity especially under inflammatory conditions. Meanwhile, these studies imply a new origination from hematopoiesis in the yolk sac before birth [10]. Recruited macrophages are mainly derived from monocytes in circulation and bone marrow derived cells (BMDC) [11]. Homeostatic control of monocyte/macrophage development is majorly driven by colony stimulating factor-1 (CSF-1, also known as M-CSF) [12]. New evidence has identified the cytokine IL-34 as a new CSF-1R ligand that influences maturation of macrophages, which is restricted to the epidermis and central nervous system [13].

### Differentiation and phenotypes of macrophages

**Fig. 1:**
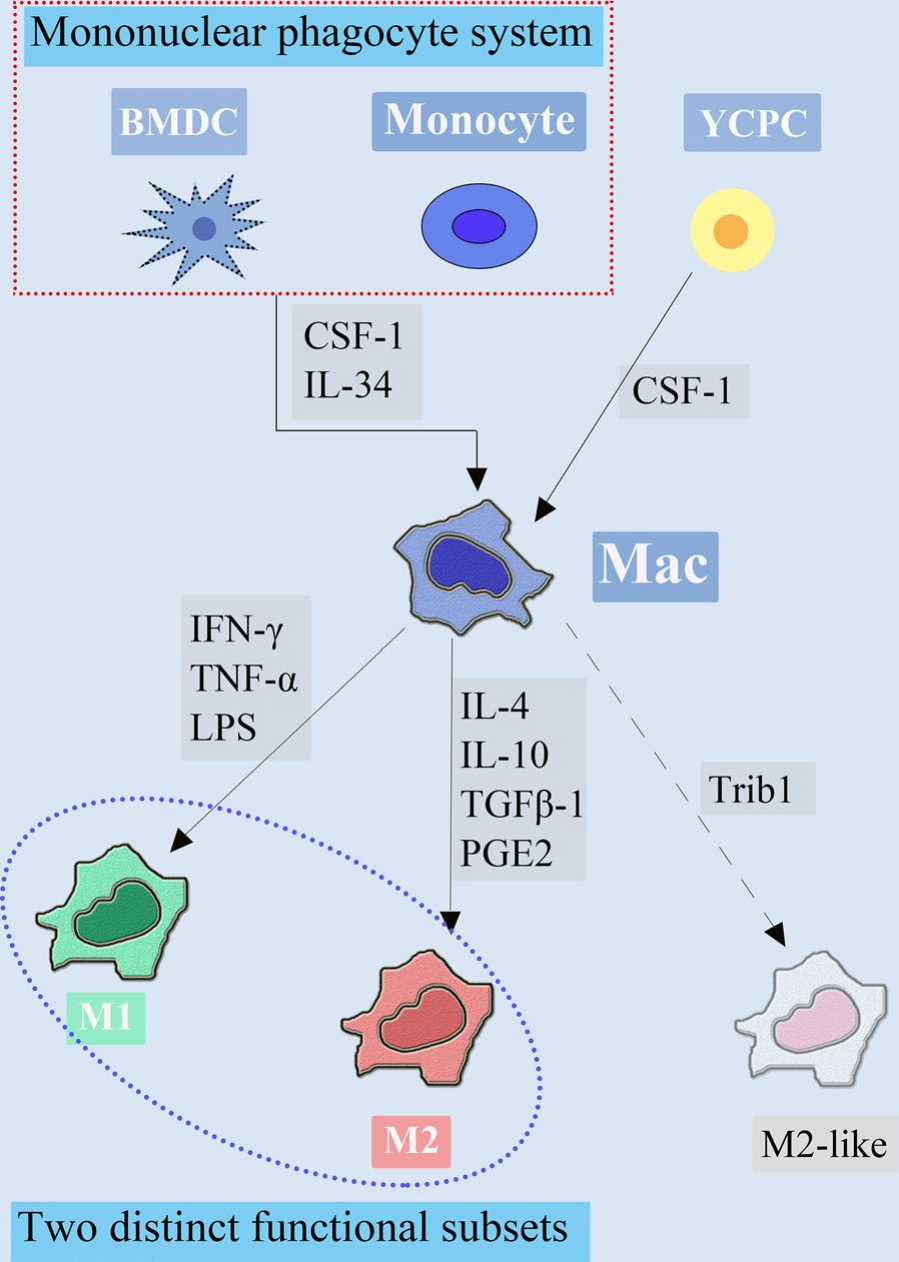
Origination and maturation of macrophages. Resident and recruited macrophages are derived from YCPC, BMDC, as well as from circulating monocytes. CSF plays crucial role in macrophages origination. Macrophages in tumor zone intimately interact with tumor cells, thereby undergoing phenotypically polarization to extremely M1 (due to the effect of IFN-γ, TNF-α and LPS, et al.) and M2 (due to the effect of IL-4, IL-10, TGFβ-1 and PGE2, et al.)

In normal tissues, macrophages could be differentiated into microglial cells in the brain, Kupffer cells in the liver, and Langerhans cells in the skin [14, 15]. In the similar manner, the phenotypes of macrophages within tumor tissues (tumor associated macrophages, TAM) also represent of innate and adaptive immune responses. Tumor related inflammation represents one of the complex hallmarks of cancer especially due to an enrichment with monocyte-derived macrophages [16]. Theoretically, in response to various stimuli, TAM could be differentiated into distinct functional subsets, extremely into M1 phenotype macrophages by Th1 (IFN-γ, TNFα, and LPS et al.), and into M2 by Th2 (IL-4, IL-10, TGFβ1, and PGE2 et al.) cytokines and immunocomplexes [17] (Fig. 1). M1 phenotype macrophages are characterized by the expression of HLA-DR and CD197, whereas M2 is typical for the high expression of CD163, CD209, CD206, and CCL2 et al. [18, 19]. In addition, potential roles of IRF4 in M1-like polarization [20] and Trib1 in tissue-resident M2-like macrophages [21] have been demonstrated, which further confirms that macrophages consist of more than two subgroups. Underlying mechanisms of TAM polarization has been demonstrated to be correlated with several major signaling pathways, including C-Jun N-terminal kinase (JNK) signaling pathway, PI3K/Akt signaling pathway, Notch signaling pathway, JAK/STAT signaling pathway, et al. [22].

### Pleiotropic but opposed activities of TAM

Herein, we would focus on the location-related distinct functional properties instead of these pathways. In normal tissues, macrophages contribute to maintaining tissue homeostasis through phases-depending mechanisms. In the initiation phase of host immune response, macrophages could generally be served as tissue sentinels via phagocytosis. Then the damaged cells and debris could be eliminated by macrophages in the phase of full inflammation. In the recovery phase, macrophages participate in the re-establishment of tissue integrity [23]. As known, tumor is partially a disease of homeostatic imbalance. Some mechanisms involved in keeping normal tissues homeostasis might also be utilized by TAM to acquire different functional phenotypes, thereafter influencing adaptive immunity towards different directions [24].

In this perspective, momentum TAM accumulated in and around tumor organs to monitor the abnormal tissue architecture, cellular compositions, and functional states [25], whereas TAM could finally be trained to perform key homeostatic functions in the maintenance of tumoral growth and progression [26]. Reasons could be offered by the issue of heterogeneity in the M1/M2. M1 phenotype macrophages are tumor-resistant due to intrinsic phagocytosis and enhanced antitumor inflammatory reactions. Contrastingly, M2 are endowed with a repertoire of tumor-promoting capabilities involving immuno-suppression, angiogenesis and neovascularization, as well as stromal activation and remodeling [27]. Functional properties of M1 and M2 would be discussed later in this review. Due to ignorance of distinct polarization of TAM, researches on exact prognostic values of TAM during cancer progression still failed to reach a consensus and remained contradictory [28]. In addition, histological distribution of TAM within cancer tissue has been analyzed and studied [29, 30], further proven to be related with corresponding functional potentials and overall influence on cancer outcomes [31, 32]. 

### Major functional properties of M1 phenotype

**Fig. 2:**
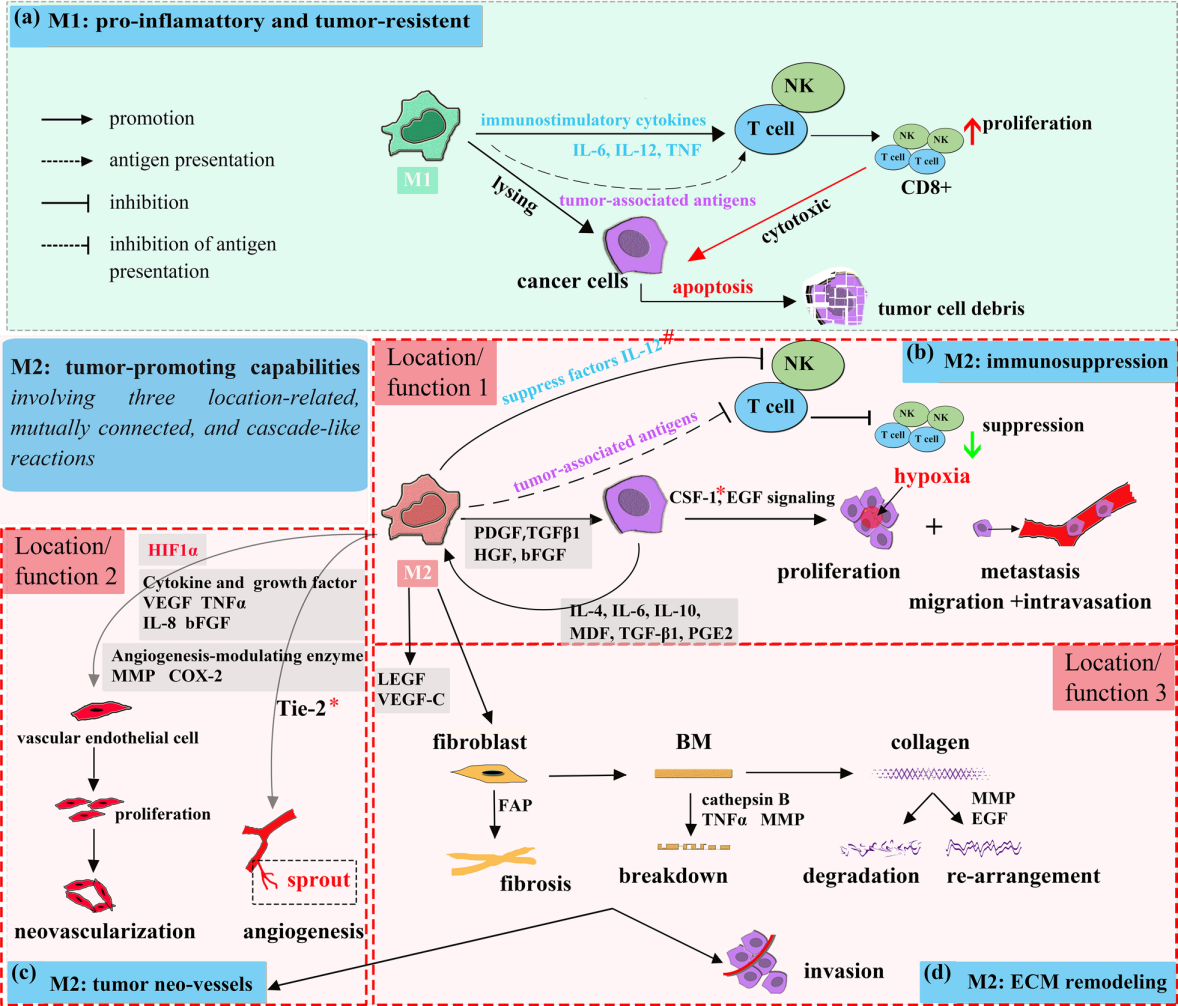
Functional characterization of M1 and M2 macrophages. **a** Generally, M1 is tumor-resistant by directly lysing tumor cells after phagocytosis, and pro-inflammatory by enhanced tumor antigen-presenting ability or by indirectly promote the proliferation of immune cells like CD8 + T cells and NK cells (due to the effect of IL-6, IL-12 and TNF, et al.). **b**–**d** Whist and however, M2 is tumor-promoting through a repertoire of mechanisms, typically summarized as immunosuppression, tumor angiogenesis and neo-vascularization, and stromal activation and remodeling. **b** In contrast to M1-related immune response, M2 obstacles host immune states (Function 1). In tumor center (defined as Location 1) growth factors (including PDGF, TGFβ, HGF, and bFGF et al.) secreted by M2 would induce proliferation and metastasis of tumor cells. As a feed-back loop, cytokines and factors (including IL-4, IL-6, IL-10, MDF, TGF-β1 and PGE2 et al.) secreted by tumor cells enhance this effect in turn. **c** In avascular and peri-necrotic areas (defined as Location 2), HIF1α induced by hypoxia or low oxygen tension, in accordance with cytokines and factors (including VEGF, TNFα, IL-8 and bFGF et al.), and angiogenesis-modulating enzymes (including MMP and COX-2 et al.) would promote neo-vascularization and induce angiogenesis (Function 2). **d** In stromal areas (defined as Location 3), M2 actively impact on CAFs differentiation, BM breakdown, and collagen degradation and re-arrangement (Function 3). These combined stromal remodeling signatures would correspondingly induce tumor neo-vessels formation and maturation, as well as tumor invasion capability. The distinct tumor-related potentials of M1/M2 should be further investigated from known mechanisms illustrated in this figure

M1phenotype macrophages have intrinsic function to trap, phagocytose, and lyse tumor cells [33]. In addition, enhanced tumor antigen presenting ability of M1 would promote other leukocytes cytotoxic functions. For instance, CD8 + T cells and NK cells could be strengthened by immuno-stimulatory cytokines (IL-6, IL-12, TNF et al.) from M1 phenotype macrophages [34]. As a result, tumor cells apoptosis would be induced at this stage. Tumor stem cells have fewer immunogenic antigens, but more vigorous proliferative and differentiative abilities. Considering the heterogeneity and tumor-intrinsic mechanisms of immune escape [35], tumor stem cells might use M1 phenotype macrophages as a natural filter to avoid being destroyed and survived to next stage [36] (Fig. 2a).

### Major functional properties of M2 phenotype

#### Immuno-modulation basing on immunoediting theory

In contrast to M1, most macrophages are switched to M2 phenotype followed by the interactions with tumor cells, which is prone to show immunosuppression potentials. Contrary, M2 act as immune-suppressor in tumor nest. On one hand, the pro-inflammatory potential of M2 phenotype macrophages are dramatically descended, due to weakened tumor antigen presenting ability [37] and the secretions of suppressor factors such as IL-12 [38]. On the other hand, M2 phenotype macrophages are educated to be tumor-promoting by releasing growth factors like PDGF, TGFβ1, HGF, and bFGF et al. [4, 39], forming a positive feed-back loop in accordance with cytokines and factors of tumor cells (IL-4, IL-6, IL-10, MDF, TGFβ1, PGE2, et al.) [40] (Fig. 2b). In fact, all macrophages are crucial for tumor proliferation at primary tumor nest, and intrigue subsequent tumor metastasis. Therefore, tumor progression could be hypothesized as stages of immune elimination, equilibrium and escape basing on M1/M2 ratios and reactions according to immunoediting theory [41].

### Angiogenesis and neovascularization

For long it has been a consensus that angiogenesis and neovascularization are essential for tumor growth and metastasis [42]. Many studies have shown that M2 phenotype macrophages are crucial to support the evolution of tumor-related vasculature, a process in which neo-vessel sprout from existing blood vessels or by proliferation, motility and accumulation of vascular endothelial cells [43]. Recent studies have emphasized the significant correlation between M2 and tumor neo-vessels with focus on perivascular and peri-necrotic areas [44]. Additionally, morphological studies have demonstrated a disorganized and collapsed characteristic of tumor neo-vessels [45]. This reason, combined with over-rapid growth of tumor cells, coordinately contribute to hypoxia zones, especially in tumor center and peri-necrotic regions. Hypoxia in these areas induces the expression of inflammatory molecules (IL-4, IL-10 et al.), thus promoting the recruitment of macrophages followed by conversion to the M2 phenotype [46]. In this case, M2 participates in a “angiogenesis cascade”, which is capable to affect the onset and maintenance of the angiogenic process, including degradation of the extracellular matrix, endothelial cell proliferation and migration, and neovascularization [47]. Molecular mechanisms underlying each phase could resulted from a variety of pro-angiogenetic cytokines and growth factors, including VEGF, TNFα, IL-8, and bFGF, as well as angiogenesis-regulating enzymes like MMP and COX-2 [48] (Fig. 2c).

### Stromal orchestration

Emerging evidence has also illustrated the significance of tumor stromal transformation in tumor progression. Herein, M2 is discussed in terms of intimate correlations with other stromal components. Actually, stromal cells around incipient tumors have a futile attempt to “destroy and repair” tumor tissues [49]. However, M2 could assist tumor cells to gain a hallmark to activate and remodel stromal features to support tumors [50].

For instance, fibroblasts differentiated from mesenchymal cells are important to tissue-repair, whereas transformed to cancer-associated fibroblasts (CAFs). In turn, CAFs also promote tumor growth and metastasis by recruiting and reprogramming macrophages into M2 phenotype [51]. In addition, M2-related enzymes promote ECM digestion and deposition, as well as the proteolysis of basement membrane (BM) and collagens surrounding tumor nests [52]. In this case, intrinsic defensive abilities of collagens are dramatically reduced. The principal factors accounted for this transformation include cathepsin B, TNFα, MMPs et al. [53].

Subsequently, degraded BM and collagen fragments may serve as chemotactic stimuli to circulating monocytes. These recruited macrophages, especially M2, could further induce the degradation and re-arrangement, enhance the stiffness of collagens, and co-orchestrate angiogenesis [54]. Thus, all these have uncovered a positive feed-back transformation displayed among M2 and other representative stromal constitutions (Fig. 2d).

### New insights into distribution pattern of macrophages

The specialization of macrophages in particular microenvironments explains their heterogeneity. In addition, the heterogeneous functional properties of macrophages would result from their location in tumor tissues [55]. There are three typical locations including tumor center, invasive front (the interface between tumor cells and stroma), and tumor stroma [56]. It has been documented that macrophages seem to be “hijacked” to perform distinct functions according to the location-related signals [22]. Briefly, M2 phenotype macrophages would be preferentially endowed with potentials to promote the motility of cancer cells in invasion areas [57], to promote metastasis in stromal and perivascular areas [31], and to stimulate angiogenesis in avascular and peri-necrotic hypoxic areas [54]. In other words, the distribution pattern of macrophages might be correlated with different cancer progression mechanisms.

Notably, we have recently found a timely novel result that the distribution pattern of macrophages could be an independent prognostic factor in gastric cancer [30]. According to relative macrophages densities in tumor nest or tumor stroma, gastric cases could be divided into nest-dominant pattern and stroma-dominant pattern. As a phenomenon, stroma-dominant pattern cases tended to have poorer survival and higher malignancy. In theory, accumulation of macrophages in tumor stroma might participate more actively in the process of stroma activation [58] and ECM remodeling, together with other stromal components including lysyl oxidase, MMP9, type IV collagen, which has been emphasized by Peng et al. [59]. In addition, Yang et al. [60] preliminarily elucidated the prognostic value of CD163+/CD68+ ratio in colorectal cancer invasive front. To make sense of macrophages polarization in different distribution patterns, studies could be conducted around macrophages phenotypes and ratios, tumor locations and biological functions in the future.

## Conclusions

In summary, M1 and M2 macrophages are functionally distinct and key modulators in host immune system against tumors. In contrast to M1, M2 mechanisms incorporate location-related, mutually connected, and cascade-like reactions. A better understanding of macrophage polarization, especially in distinct locations could make sense for tumor progression and guide therapy in the future.

## Data Availability

Not applicable.

## Abbreviations

BM: Basement membrane
BMDC: Bone marrow derived cell
bFGF: Basic fibroblast growth factor
CAFs: Cancer-associated fibroblasts
COX: Cyclooxygenase
CSF: Colony stimulation factor
ECM: Extracellular matrix
EGF: Epidermal growth factor
IFN-γ: Interferon-γ
IL: Interleukin
LEGF: Lymphatic endothelial growth factor
LPS: Lipopolysaccharide
Mac: Macrophages
MDF: Myocardial depressant factor
MMP: Matrix metallo proteinases
NK: Natural killer cell
PGE2: Prostaglandin E2
TAM: Tumor associated macrophages
TNF-α: Tumor necrosis factor-α
VEGF: Vascular endothelial growth factor
YCPC: Yolk sac progenitor cell
